# Percutaneous Internal Ring Suturing for the Minimally Invasive Repair of Congenital Inguinal Hernia in Neonates: A Retrospective Cohort Study

**DOI:** 10.7759/cureus.36180

**Published:** 2023-03-15

**Authors:** David T Thomas, Serkan Tulgar

**Affiliations:** 1 Department of Pediatric Surgery, Maltepe University Faculty of Medicine, Istanbul, TUR; 2 Department of Anesthesiology and Reanimation, Samsun University Faculty of Medicine, Samsun, TUR

**Keywords:** percutaneous internal ring suturing, pediatric, child, neonate, laparoscopy, herniorrhaphy, contralateral patent processus vaginalis, inguinal hernia

## Abstract

Introduction

Inguinal hernia (IH) repair is among the most frequently performed surgical operations in children. While open herniorrhaphy has been the gold standard surgical method of choice, the popularity of laparoscopic repair has sharply risen over the past two decades. Although a wide range of literature on the use of laparoscopy for IH repair in children exists, data regarding neonates, an especially delicate group of children, is limited to only a few studies. This study aims to evaluate the surgical, anaesthetic, and follow-up data of term neonates undergoing percutaneous internal ring suturing (PIRS) for IH repair in order to determine if it is a viable option for this specific patient population.

Materials and methods

This single-centre retrospective cohort included all children who underwent PIRS for IH repair during an 86-month period between October 2015 and December 2022. Patients’ gender, gestational age at birth, age and weight at surgery, side of IH at diagnosis, per-operative findings (presence of contralateral patent processus vaginalis (CPPV)), surgical time, time under anaesthesia, follow-up time, and follow-up findings were collected from an electronic database and analysed. The primary outcome measures were surgical time, rate of recurrence, and presence of CPPV and the secondary outcome measures were anaesthesia time and the rate of complications.

Results

During the study period, 34 neonates (23 male and 11 female) underwent laparoscopic repair for IH using the PIRS technique. Average age and weight at surgery were 25.2 ± 3.2 (20-30) days and 3530.4 ± 293.6 (3012 - 3952) gm, respectively. IH was detected on the right side in 19 (55.9%), on the left side in 12 (35.3%), and bilaterally in three (8.8%) patients at their presenting physical examination. Nine patients (26.5%) were found to have CPPV perioperatively, which were all repaired simultaneously. The average surgical time was 20.3 ± 4.5 minutes for unilateral and 25.8 ± 4.0 minutes for bilateral IH repair (p<0.01). On the contrary, the average time under anaesthesia was 33.2 ± 4.5 minutes for unilateral and 33.5 ± 4.9 minutes for bilateral IH repair, but the difference was not statistically significant (p>0.05). No early postoperative complications were observed. The average follow-up time was 27.6 ± 14.4 (range: 3-49) months. Recurrence was seen in one patient (2.9%) and umbilical incision granuloma was observed in two (5.9%) patients.

Conclusion

Surgical times, anaesthesia times, complication rates, recurrence rates, and rate of CPPV in neonates undergoing PIRS are similar to those in older children and comparable to those of open herniorrhaphy and other laparoscopic techniques. Despite the suspicion that the rate of CPPV would be higher in neonates, we found that it is similar to that in older children. We conclude that PIRS is a viable option for the minimally invasive repair of IH in neonates.

## Introduction

Inguinal hernia (IH) repair is among the most frequently performed surgical operations in children. It occurs in between 0.8% and 4.4% of all children [1]. In contrast to adults, the vast majority of IHs in newborns are congenital and caused by a patent processus vaginalis (PPV). Open herniorrhaphy remains the surgical method of choice and is associated with high success rates and minimal complications. 

Laparoscopic repair of IH was initially described in 1997, when El Gohary reported a technique where the inverted hernia sac was ligated using an endoloop suture in girls [2]. Since then, numerous intracorporeal and extracorporeal approaches have been described for the laparoscopic repair of IHs in children. All techniques maintain the fundamental premise of surgical repair for paediatric IHs: high ligation of the sac.

Patkowski et al. originally described percutaneous internal ring suturing (PIRS) in 2006, as a minimally invasive treatment for managing IHs in children. This extracorporeal technique uses a laparoscope for visualisation, while a readily available intravenous cannula needle is used to deliver a suture that ligates the hernia sac at the level of the internal ring [3,4]. We have previously reported our surgical and anaesthesia-related experience using PIRS in children over one year of age [5,6].

Neonates are an especially delicate group of children. The timing of IH repair in this group of children remains a topic of debate. Delayed repair is associated with complications such as incarceration, yet early repair has been associated with high risk and may be technically challenging [7].

Although a wide range of literature on the use of laparoscopy for IH repair in children exists, data regarding neonates is limited to only a few studies. This study aims to evaluate the surgical, anaesthetic, and follow-up data of term neonates undergoing PIRS to determine whether PIRS is a viable option for the repair of IH in neonates.

## Materials and methods

Patients

Our retrospective cohort consisted of all children under 30 days of age who underwent laparoscopic surgery using the PIRS technique for IH at Maltepe University Faculty of Medicine Hospital, Istanbul, Türkiye, during a period of 86 months between October 2015 and December 2022. A single paediatric surgeon performed all procedures (DTT). Prospectively collected data were retrospectively analysed.

Children who underwent PIRS repair for IH that were ≥38 gestational weeks of age at birth and who were aged < 30 days at surgery were included in the study. To avoid confounding factors, children born <38 gestational weeks, those with metabolic or systemic diseases that could affect surgery or anaesthesia time, and those who required neonatal intensive care were excluded.

Surgical time (primary outcome) and anaesthesia time (secondary outcome) were used for sample size calculation, as these times are important factors in neonatal surgery. Based on the results of our previously published data [5], the minimum required sample size for this study was calculated to be 27 patients, using alpha and beta of 0.05.

Techniques and definitions

The first author of this paper has routinely and exclusively employed PIRS to repair IHs and communicating hydroceles in paediatric patients since 2013 and has reported findings, including data on the learning curve for PIRS, previously [5]. 

Inhaled sevoflurane was used for induction and maintenance of anaesthesia. The neonates were ventilated with 8, then 5, then 3 MAC (minimum alveolar concentration) of sevoflurane, respectively, during induction; 3.2 MAC sevoflurane was used during maintenance. The patients were administered a mixture of O_2_ and air. Manual ventilation was performed with a tidal volume of 8-10 ml/kg. No opiates were used. 

In accordance with Patkowski et al.'s prior description [5], the PIRS method was performed on all patients as follows: Neonates were given general anaesthesia and their airways were maintained with endotracheal intubation. After endotracheal intubation, an orogastric 8 Fr or 10 Fr feeding tube was placed for gastric decompression. Following local anaesthesia infiltration and a small umbilical incision, a Veress needle was used to insufflate the abdominal cavity. For all procedures, a single 5 mm port, a 5 mm 30° telescope and 8 mmHg insufflation pressure were utilized. No patient required higher insufflation pressure. 

A 2 mm stab incision above the internal ring was performed after observing the internal inguinal ring and the PPV. An 18 G intravenous cannula needle equipped with a 5/0 nonabsorbable monofilament suture was passed on one side of the internal ring, continually entering and exiting the peritoneal cavity. The loop was extended into the peritoneal cavity from the farthest opposing side allowed by the needle. The 18 G intravenous cannula needle was then introduced on the opposite side of the internal ring, entering and exiting the peritoneal cavity several times before returning to the farthest opposite side allowed by the needle. The needle was then placed through the previously created loop and threaded with a nonabsorbable 4/0 monofilament suture. After removing the needle, the loop was retracted, catching the second suture and delivering it from the stab wound. Any gas in the hernia sac was removed by manual reduction. Subsequently, the internal ring was obliterated by tying the suture subcutaneously. The repair was considered successful if the hernia sac was observed to be completely occluded on laparoscopy, and if there was no re-insufflation of the hernia sac (lump in the inguinal area or scrotum). Absorbable 4/0 sutures were used to close the fascia and subcutaneous layers of the umbilical wound, and sterile braided adhesive strips were used to close the skin at the umbilical and inguinal puncture incisions. 

Feeding was commenced after the second postoperative hour in all patients. At the 12th postoperative hour, patients were discharged if they were feeding normally, had no occurrences of apnea, and were pain-free. Rectal paracetamol (10 mg/kg) was administered to manage pain for the first 48 hours following surgery both in the hospital and at home.

All children received a follow-up visit and physical examination on the postoperative seventh day. Parents were instructed to return for further examination if a recurrence was suspected (lump in groin area or scrotum/labia) or if there were any other concerns (bleeding, redness, oedema, or mass/nodule in the areas of the incisions).

Collected data & statistical analysis

Patients’ gender, gestational age at birth, age and weight at surgery, side of IH at diagnosis, preoperative findings including presence of contralateral PPV (CPPV), surgical time, anaesthesia time, follow-up time, and follow-up findings were collected from an electronic database and analysed.

Gestational age was calculated according to the last menstrual period of the mother and measured as weeks. Age and weight at surgery were measured in days and grams, respectively. The side of IH at diagnosis was recorded as left, right, or bilateral. CPPV was defined as the existence of a peritoneal opening at the internal inguinal ring that extended into the inguinal canal on the opposite side of the planned surgery. The duration of the surgery was measured from the commencement of skin preparation to the completion of wound dressings. Anaesthesia time was defined as the period between induction and a modified Aldrete score [8] of nine or more. Surgical and anaesthesia times were measured in minutes.

All parents were given information about the surgery at their outpatient consultation. The parents were informed about the potential of switching from laparoscopic to open surgery.

Google Sheets (Google LLC., Mountain View, California, United States) and tools found at openepi.com were used to perform statistical analysis. We presented continuous data as means and standard deviations and categorical data as numbers with percentages. The Shapiro-Wilk test was used to test normality of data. As data was found to be normally distributed, surgical and anaesthesia times were compared using the student's t-test. For all other comparisons, the Mann-Whitney U test was utilized. Statistical significance was accepted as p<0.05.

Ethics

The Maltepe University Clinical Research Ethics Committee approved the study (approval nunber: 2023/900/08, dated January 24, 2023). The trial was registered with clinicaltrials.gov (NCT05702710). The study was conducted in accordance with the Declaration of Helsinki. Each parent completed an informed consent form for the surgical procedure. All parents also gave their consent for their children's data to be used and published in scientific studies.

Outcome measures

The primary outcome measures were surgical times, rate of recurrence, and presence of CPPV. The secondary outcome measures were anaesthesia time and the rate of complications.

## Results

During the study period, 34 neonates underwent laparoscopic repair for IH using the PIRS technique. The characteristics of neonates included in the study are shown in Table 1.

**Table 1:** Characteristics and follow-up time of neonates included in the study M: male; F: female; IH: inguinal hernia

Characteristic	Results
Gender (M/F), n (%)	23 (67.6%) / 11 (32.1%)
Gestational Age at birth (weeks), median ± SD	38.6 ± 1.3 (37-40)
Age at surgery (days), median ± SD	25.2 ± 3.2 (20-30)
Weight at surgery (kg), median ± SD	3530.4 ± 293.6 (3012-3952)
Side of IH at presenting physical examination, n (%)	Right: 19 (55.9%) Left: 12 (35.3%) Bilateral: 3 (8.8%)
Follow-up time (months), median ± SD	27.6 ± 14.4 (3 - 49)

Table 2 demonstrates the results of our primary and secondary outcomes, as well as their comparison for patients undergoing unilateral and bilateral PIRS.

**Table 2:** Primary and secondary outcome results for all patients, as well as a comparison of surgical time and anaesthesia time between patients undergoing unilateral and bilateral PIRS ^1^: Student's t-test CPPV: contralateral patent processus vaginalis; PIRS: percutaneous internal ring suturing

Type of Outcome	Outcome	All patients	Unilateral	Bilateral	p ^1^
Primary	Presence of CPPV, n (%)	9 (26.5%)			
	Recurrance during follow-up, n (%)	1 (2.9%)			
	Surgical Time (minutes), median ± SD	22.3 ± 5.1	20.3 ± 4.5	25.8 ± 4.0	<0.01
Secondary	Anaesthesia time (minutes), median ± SD	33.3 ± 4.7	33.2 ± 4.5	33.5 ± 4.9	>0.05
	Rate of complication, n (%)	2 (5.9%)			

As expected, average surgical time was statistically significantly higher in neonates undergoing bilateral IH repair (p<0.01). However, time under anaesthesia was nearly exactly the same regardless of whether a patient underwent unilateral or bilateral IH repair (p>0.05).

## Discussion

Although laparoscopic surgery is utilised in neonates, data regarding the use of laparoscopy for IH repair in this group of especially delicate children is sparse. In a recent review that evaluated 32 studies (three randomised controlled trials, 11 cohort studies, 13 cross-sectional studies, and five case studies) of laparoscopic repair of IH in children, only six of the studies were reported to include neonates [9]. Of the six studies, none reported how many of the study population were neonates, and the average age of children in all studies was >1 year. To the best of our knowledge, this is the first study to solely evaluate data of neonates undergoing laparoscopic IH repair.

The use of laparoscopy for the repair of IH remains a topic of debate, although evidence of the various advantages over open surgery has been increasing in recent years. In a meta-analysis published in 2022, the data of 64,733 children undergoing open repair were compared with 26,920 children undergoing laparoscopic repair for IH [10]. The authors reported that the operative time for laparoscopic repair in unilateral and bilateral IHs was lower in laparoscopic surgery, but that operative time was longer in females with unilateral hernia when compared to open surgery. The overall operative times were found to be similar. The recurrence rates were also similar between the groups; however, the rate of complications was less in laparoscopic surgery. The overall rate of CPPV was reported as 39.6%. The authors concluded that laparoscopic repair has additional advantages over open repair in not just selected cases but in all children.

Various intracorporeal and extracorporeal methods exist for the laparoscopic repair of IHs in children. Most techniques use two or three ports to perform high ligation with intracorporeal suturing, which requires some experience and is time-consuming [11]. PIRS on the other hand utilises only one umbilical port for visualisation of the internal ring and PPV, making it easier and less expensive when compared to other approaches, although to our knowledge a cost-effectiveness study comparing different techniques has not yet been performed. In neonates, extracorporeal techniques such as PIRS could be advantageous, as intracorporeal suturing may be more challenging considering the relatively small intra-abdominal space. This is why we choose to utilize PIRS both in our daily clinical practice and as a topic of this study.

Previously, we reported our experience of 250 procedures in children >1 year of age in which PPV was present on the right in 53.1%, left in 35.2%, and bilaterally in 11.7% of patients [5]. The rate of contralateral PPV was 16.4%. The mean surgical time was 14.3 minutes for unilateral and 20.4 minutes for bilateral PIRS, and the mean anaesthesia time was 33.6 minutes for unilateral and 39.1 minutes for bilateral PIRS. The recurrence rate was 1.4% and the complication rate was 2.8%. In the current study of neonates, the mean surgical time was determined as 20.4 minutes for unilateral and 25.8 minutes for bilateral PIRS and anaesthesia time as 33.2 minutes for unilateral and 33.5 minutes for bilateral PIRS. The rate of recurrence and complications were 2.9% and 5.9%, respectively. When data from these two studies were compared, the difference in complication and recurrence rates were not statistically significant (p=0.449 and p=0.298, respectively).

The first primary outcome of our study was average surgical time. When we compare the findings of our current study of neonates with our previous study of children >1 year old [5], our surgical times appear to be higher in neonates (20.3 vs 14.3 for unilateral and 25.8 vs 20.4 for bilateral repair.) On the other hand, in a study of children <3 months of age undergoing PIRS [12], the mean surgical time of 85 children was reported as 24 minutes for unilateral and 33 minutes for bilateral PIRS, similar to our findings. Patkowski et al. also reported their experience with PIRS in children <3 months of age, with an average surgical time of 23 minutes for unilateral and 26 minutes for bilateral hernias [11]. Therefore, our surgical times appear to be similar to those reported in the literature.

Our first secondary outcome was anaesthesia time. Time spent under anaesthesia is especially significant in neonates. In our study, we found that patients undergoing bilateral IH repair had similar anaesthesia times as those undergoing unilateral repair (33.2 vs 33.5 minutes), despite a statistically significant and expected increase in surgical time for bilateral IH repair (25.8 vs 20.3 minutes). Patkowski et al. reported an overall anaesthesia time of 48 minutes [11], which is significantly longer when compared to our data. In a study comparing single-incision laparoscopic herniorrhaphy (SILH) in newborns and infants, the average time under anaesthesia was reported to be 80.0 ± 14.5 minutes for newborns and 76.3 ± 12.9 minutes for infants (p=0.07) [13]. Average anaesthesia times in our study appear to be lower than those in the literature. Our current data suggest that laparoscopic repair of IH in newborns is not associated with increased anaesthesia times.

The remaining two primary outcomes of our study were the rate of recurrence and the presence of CPPV. Recurrence is reported to occur in 0.3-10.9% of patients undergoing open IH repair [14]. In a literature review of 10183 cases, recurrence after laparoscopic repair was reported to be 0.8% [9]. For PIRS, two studies that included 204 and 124 patients, respectively, reported no recurrences at all [11,15]. In our study, which consisted of only neonates, recurrence was observed in only one out of 34 patients (2.9%). Our rate appears to be higher as a percentage compared to these studies, but we believe this to be due to our study size. On the other hand, one study of 240 patients reported a much high recurrence rate of 18.6% for PIRS [16]. Additionally, a study that compared open vs laparoscopic repair reported the recurrence rate of laparoscopic repair was higher when compared to open surgery (3.8% vs 1.4%) [14].

One of the most significant advantages of laparoscopic IH repair is the detection of a CCPV, potentially saving the child from a second surgical procedure. In our current study, CPPV was observed in nine patients (26.5%). This rate is higher than our previously published findings on children >1 year of age (16.4%) [5]. We performed a statistical analysis to compare both data and found that this difference is not statistically significant (p=0.07). In a recent study of 276 children undergoing PIRS, CPPV was observed in 19.2% [17]. Reporting their 20-year experience with laparoscopic IH repair, Esposito et al. reported a CPPV rate of 41% amongst 1300 children [18]. Ho et al. reported an even higher rate of CCPV (73.5%) in their series of 113 patients [15]. It could be suggested that CCPV may be observed at a higher rate in neonates, as the exact timing of closure of PPV is unclear. However, despite the limited number of patients, our current findings suggest that the rate of CPPV is not higher in neonates when compared to older children. Furthermore, considering the high rate of CPPV reported in the studies mentioned above, the question also remains regarding what percentage of CPPV detected on laparoscopy would indeed become symptomatic and require surgery for metachronous IH in the future.

Complications of laparoscopic IH have generally been reported to be under 1%. Grech et al. reported complications in 0.7% of patients in a series of over 10,000 children [9]. These complications included testicular atrophy, port-related complications, wound infection and others. We observed umbilical incision site granuloma in two patients (5.9%). There is no obvious reason why a higher complication rate should be observed in neonates and therefore we believe that the higher percentage of complications seen in our study when compared to other studies is due to our low number of cases.

The findings of this study should be interpreted in the context of its limitations. The retrospective design of the study, the lack of a comparison group consisting of neonates undergoing open surgery, and the small number of patients are the major drawbacks of this study. A multicentre, prospective study with standardised data collection and long follow-up protocols are necessary to demonstrate the efficacy and non-inferiority/superiority of PIRS when compared to open or other laparoscopic techniques in neonates.

## Conclusions

Our study has demonstrated that PIRS is a viable laparoscopic technique for the repair of IH in neonates. Surgical times, anaesthesia times, complication rates, and recurrence rates of neonates undergoing PIRS are similar to those in older children undergoing either PIRS, other laparoscopic IH repair techniques, or open herniorrhaphy. Despite the suspicion that the rate of CPPV would be higher in neonates, we found that it is similar to that of older children.

Although growing evidence suggests that PIRS is advantageous over other laparoscopic or open techniques in terms of our primary and secondary outcomes in older children, to the best of our knowledge, this is the first study to evaluate and demonstrate this data in a study population consisting solely of neonates.
